# CDK7 inhibition augments response to multidrug chemotherapy in pancreatic cancer

**DOI:** 10.1186/s13046-022-02443-w

**Published:** 2022-08-10

**Authors:** Siyuan Zeng, Bin Lan, Xiaofan Ren, Shuman Zhang, Daniel Schreyer, Markus Eckstein, Hai Yang, Nathalie Britzen-Laurent, Andreas Dahl, Debabrata Mukhopadhyay, David Chang, Isabella Kutschick, Susanne Pfeffer, Peter Bailey, Andrew Biankin, Robert Grützmann, Christian Pilarsky

**Affiliations:** 1 Department of Surgery, Universitätsklinikum Erlangen, Translational Research Center, Schwabachanlage 12, 91054 Erlangen, Germany; 2 Wolfson Wohl Cancer Research Centre, Institute of Cancer Sciences, University of Glasgow, Garscube Estate, Glasgow, Scotland, UK; 3 Institute of Pathology, Universitätsklinikum Erlangen, 91054 Erlangen, Germany; 4 DRESDEN-Concept Genome Center a DFG NGS Competence Center, TU Dresden, 01307 Dresden, Germany; 5 Departments of Biochemistry and Molecular Biology, Mayo Clinic College of Medicine and Science, Jacksonville, USA; 6 Wolfson Wohl Cancer Research Centre, Institute of Cancer Sciences, University of Glasgow, Glasgow, Scotland, UK; 7 West of Scotland Pancreatic Unit, Glasgow Royal Infirmary, Glasgow, UK

**Keywords:** CRISPR-Cas9, CDK7, Gemcitabine, Paclitaxel, FOLFIRINOX, THZ1

## Abstract

**Background:**

Pancreatic ductal adenocarcinoma (PDAC) is an aggressive cancer with a dismal prognosis. Although combined treatment with gemcitabine and albumin-bound paclitaxel has improved the prognosis of PDAC, both intrinsic and acquired chemoresistance remain as severe hurtles towards improved prognosis. Thus, new therapeutic targets and innovative strategies are urgently needed.

**Methods:**

In this study, we used the KPC mouse model-derived PDAC cell line TB32047 to perform kinome-wide CRISPR-Cas9 loss-of-function screening. Next-generation sequencing and MAGeCK-VISPR analysis were performed to identify candidate genes. We then conducted cell viability, clonogenic, and apoptosis assays and evaluated the synergistic therapeutic effects of cyclin-dependent kinase 7 (CDK7) depletion or inhibition with gemcitabine (GEM) and paclitaxel (PTX) in a murine orthotopic pancreatic cancer model. For mechanistic studies, we performed genome enrichment analysis (GSEA) and Western blotting to identify and verify the pathways that render PDAC sensitive to GEM/PTX therapy.

**Results:**

We identified several cell cycle checkpoint kinases and DNA damage-related kinases as targets for overcoming chemoresistance. Among them, CDK7 ranked highly in both screenings. We demonstrated that both gene knockout and pharmacological inhibition of CDK7 by THZ1 result in cell cycle arrest, apoptosis induction, and DNA damage at least predominantly through the STAT3-MCL1-CHK1 axis. Furthermore, THZ1 synergized with GEM and PTX in vitro and in vivo, resulting in enhanced antitumor effects.

**Conclusions:**

Our findings support the application of CRISPR-Cas9 screening in identifying novel therapeutic targets and suggest new strategies for overcoming chemoresistance in pancreatic cancer.

**Supplementary Information:**

The online version contains supplementary material available at 10.1186/s13046-022-02443-w.

## Background

PDAC is the fourth leading cause of cancer-related mortality globally and is expected to overtake lung cancer as the second leading cause of cancer-related death by 2030 [1]. According to the Global Cancer Statistics 2020, a total of 495,773 new cases of pancreatic cancer (PC) and 466,003 related deaths were recorded worldwide in 2020, with the mortality rate almost as high as the incidence rate [2]. Despite advances in the treatment of pancreatic cancer, the 5-year survival rate remains at 9% [3, 4]. The poor prognosis associated with PC is primarily due to the low rate of early detection, the cancer’s rapid progression, and limited treatment options [5–7]. Currently, the first-line chemotherapy regimens for patients with advanced or metastatic pancreatic cancer are combinations of 5-fluorouracil/leucovorin with irinotecan and oxaliplatin (FOLFIRINOX) or gemcitabine plus nanoparticle albumin-bound paclitaxel, which never have been compared head-to-head in a clinical trial. Real-world retrospective analyses revealed a better overall survival rate of younger patients receiving FOLFIRINOX than of patients receiving gemcitabine plus nab-paclitaxel [8, 9]. However, both schemes are hampered by a high rate of non-responders [10, 11]. Thus, unraveling the molecular mechanisms that limit the efficacy of chemotherapy and new strategies that increase the efficacy of chemotherapy in PDAC are urgently required.

CRISPR-Cas9-based screening of small-guide RNA (sgRNA) libraries has evolved into a potent tool for elucidating the molecular processes underlying drug response and discovering novel targets for chemotherapy [12–14]. As major regulators at nearly every developmental stage, protein kinases modulate most of the signal transmission that takes place in eukaryotic cells [15–17]. They therefore represent interesting targets for CRISPR-Cas9 screens. The dysregulation of protein kinases is associated with a number of carcinogenic processes, including chemoresistance [18, 19]. In PDAC, oncogenic KRAS drives the transcriptional expression of PI3K (phosphatidylinositol 3-kinase)/AKT (alpha serine/threonine-protein kinase)/mTOR (mammalian target of rapamycin) and RAF (rapidly accelerated fibrosarcoma)/MEK/MAPK (mitogen-activated protein kinase), which have been shown to regulate cancer cell proliferation and survival as well as resistance to chemotherapy [20–24]. The combination of protein kinase inhibitors with chemotherapeutic drugs could therefore prove a promising strategy for PDAC. To date, several targeting inhibitors have been evaluated in clinical trials in patients with metastatic PDAC, and have not been found to be of significant benefit compared to single agents [25–27]. The development of novel and effective targeted inhibitors to improve the efficacy of existing treatments with synergistic effects has become a prominent research area. Thus, CRISPR-Cas9 opens up new avenues for the development of novel targeted inhibitors for overcoming chemoresistance.

We conducted CRISPR-Cas9 screening of protein kinases and PDAC-related genes to analyze their role in chemoresistance of PDAC cells to GEM and PTX. We identified several candidate genes enriched in DNA damage repair, cell cycle progression, and cell apoptosis. The screening results were verified by CRISPR-Cas9 knockout of multiple target gene candidates. Deletion of CDK7 inhibited PDAC cell proliferation, promoted apoptosis, and suppressed cell cycle progression in human and murine cell lines, suggesting that CDK7 expression is required for chemoresistance.

Previous research (Fig. S1A) revealed that CDK7 is involved in cell cycle regulation, transcription initiation, and elongation [28, 29]. Numerous covalent CDK7 inhibitors have also been discovered, most prominently THZ1. THZ1 promotes apoptosis by inhibiting the expression of B-cell lymphoma 2 (Bcl-2) and MCL1 Apoptosis Regulator (BCL2 Family Member) [30]. THZ1 also inhibits tumor stem cells in chemoresistant uroepithelial carcinoma through the hedgehog signaling pathway [31]. In highly aggressive PDAC, CDK7 inhibition resulted in significant downregulation of gene transcription and preferential inhibition of the mitotic cell cycle [32]. Ultimately, targeted inhibition of CDK7 overcomes chemoresistance primarily by suppressing downstream gene transcription, inhibiting mitosis, and thus promoting cell cycle arrest. A rising number of studies have recently focused on the role of CDK7 in cancer, but we observed limited data on the role of CDK7 inhibitors in combination with chemotherapy, particularly in pancreatic cancer. We discovered through a series of in vivo and in vitro experiments that CDK7 inhibitors not only have anticancer effects alone, but also act as enhancers of conventional chemotherapy combinations and as re-sensitizers following chemotherapy failure. More crucially, we present a theoretical foundation and strategy for developing novel targeted inhibitors by combining CRISPR-Cas9 screening with organoid culture and pancreatic orthotopic injection models. In terms of pharmacological mechanisms, although the pharmacological effects of GEM and PTX as conventional chemotherapeutic agents in pancreatic cancer are well established, as we summarize in Fig. 6C, targeted inhibition of CDK7 exhibits substantial synergistic effects with GEM and PTX combination chemotherapy, revealing new prospective therapeutic options for the treatment of PDAC.

## Methods

### Cell culture and reagents

Human pancreatic cancer cell lines PANC-1 (ATCC, Cat. #CRL-1469, RRID: CVCL_0480), AsPC-1 (ATCC, Cat. #CRL-1682, RRID: CVCL_0152), BxPC-3 (ATCC, Cat. #CRL-1687, RRID: CVCL_0186), MIA PaCa-2 (ATCC, Cat. # CRM-CRL-1420, RRID: CVCL_0428), SUIT-2 (JCRB, Cat. #JCRB1094, RRID: CVCL_3172), TKCC-10, and Mayo4636 and the murine pancreatic cancer cell line TB32047 are listed in Table S1. PANC-1, AsPC-1, BxPC-3, and MIA PaCa-2 were purchased from American Type Culture Collection (ATCC). PANC-1, AsPC-1, and BxPC-3 were cultured in RPMI-1640 medium (Cat. #21,875–034, Gibco) supplemented with 10% FBS (Cat. #A3160401, Gibco). MIA PaCa-2 cells were cultured in DMEM (Cat. #30,966–021, Gibco) medium with 10% FBS and 2% horse serum (Cat. #16,050–130, Gibco). SUIT-2 cells were purchased from Japanese Collection of Research Bioresources Cell Bank (JCRB) and cultured in MEM (Cat. #31,095–029, Gibco) supplemented with 10% FBS. Patient-derived cell lines (PDCLs) TKCC-10 were cultured in conditions specifically formulated as previously described [33]. Mayo4636 were cultured in DMEM/F12 (Cat. #11,320–074, Gibco) supplemented with 10% FBS. Primary murine pancreatic cancer cell line TB32047 was kindly provided by David Tuvenson, Cold Spring Harbor Laboratory (CSHL), and cultured in DMEM medium supplemented with 10% FBS. HEK293TN (BioCat, Cat# LV900A-1-GVO-SBI, RRID: CVCL_UL49) cells were obtained from BioCat and maintained in DMEM supplemented with 10% FBS. All cell lines were grown in monolayers at 37 °C in a humidified environment containing 5% CO2 and tested for mycoplasma contamination. Human cell line identity was confirmed by STR analysis. All cells were harvested by 0.25% Trypsin–EDTA (Cat. #25,200–072, Gibco). DNA fingerprinting by highly polymorphic short tandem repeat (STR) analysis was used to authenticate cells, and mycoplasma testing was performed on a regular basis to ensure cells were free from mycoplasma contamination.

The chemotherapeutic drugs gemcitabine (GEM, 40 mg/mL) and paclitaxel (PTX, 6 mg/mL) were purchased from the pharmacy of the University Hospital, Erlangen in ready-made solutions, and nab-paclitaxel (Pazenir, 5 mg/ml) was purchased from Ratiopharm GmbH, Germany. The small-molecule inhibitor targeting CDK7 (THZ1, Cat. #HY-80013) was purchased from MedChemExpress.

### sgRNA library and lentivirus production

The pooled sgRNA library (Mouse Brie kinome pooled library) targeting murine kinome was a gift from John Doench and David Root (Addgene, RRID: Addgene_75316) with additional sgRNAs for genes involved in pancreatic cancer [34] (library files, please see Supplementary Table.zip). The lentivirus was produced as described previously [28], and the plasmids are listed in Supplementary Table S2. Briefly, three T175 flasks of HEK293TN cells were plated at 30% confluence in antibiotic-free DMEM supplemented with 10% FBS. Transfection was performed with Lipofectamine™ 3000 Transfection Reagent (Cat. # L3000001, Invitrogen). For each flask, 13.8 μg of pooled-sgRNA library, 9.2 μg of pMDLg/pRRE (Cat. #12,251, Addgene), 4.6 μg of pRSV-REV (Cat. #12,253, Addgene), 4.6 μg of pMD2.G (Cat. #12,259, Addgene), 64.4 μL P3000 Enhancer Reagent, and 129 μL of Lipofectamine™ 3000 diluted in OptiMEM (Cat. #31,985,070, Gibco) were mixed and added to the HEK293TN cells. pMDLg/pRRE (Addgene; RRID: Addgene_12251), pRSV-REV (Addgene; RRID: Addgene_12253), and pMD2.G (Addgene, RRID: Addgene_12259) were gifts from Didier Trono [35]. Medium was changed 6 h post-transfection, and virus-containing medium was collected 24 h later and filtered through a 0.45 μm strainer. TB32047-Cas9 cells were produced by transfection with lentivirus generated from the lentiCas9-Blast plasmid which was a gift from Feng Zhang [36].

### CRISPR-Cas9 knockout screening with protein kinases library

The CRISPR screen was performed essentially as described previously [12]. Briefly, 1.8 million TB32047 cells were transduced in triplicates with kinome-wide sgRNA lentivirus (MOI ∼0.3). Cells were then selected with puromycin (10 μg/mL) for 3 days. Ten million transfected cells were harvested as a baseline (D0 treatment). The remainder of the surviving cells were divided into three groups (T75 flask/group) following treatment with vehicle (PBS), gemcitabine (IC90 = 50 nM), or paclitaxel (IC70 = 10 nM) for 21 days. At least 10 million cells for each group were collected for DNA sequencing at the end of the treatment.

### Genomic DNA sequencing and data analysis

The isolation of genomic DNA (gDNA) was performed with a NucleoSpin Blood L kit (Cat. #740,954.20, Macherey–Nagel), followed by the PCR procedure to amplify sgRNAs. For the PCR reaction, 10 µg DNA was used in order to achieve 300X coverage. For each sample, Q5 master mix was used to perform two separate 100 µl reactions with 5 µg of genomic DNA in each reaction. All PCR primers are listed in Supplementary Table S2 and were synthesized by Eurofins. The resulting PCR amplicons from the PCR reactions were purified and sequenced by NovaSeq 6000 (Illumina). The number of reads for each sgRNA was quantified and normalized to total reads of all sgRNAs using MAGeCK-VISPR software, a comprehensive quality control (QC), analysis, and visualization workflow for CRISPR screens. MAGeCK-VISPR comprises a maximum-likelihood algorithm to identify essential genes simultaneously under multiple sets of conditions, as well as a set of QC measures to assess the quality of an experiment. To deconvolute different effects, the algorithm leverages a generalized linear model and uses expectation maximization to iteratively estimate sgRNA knockout efficiency and gene essentiality [36]. The result of the sequencing can be found in the EBI database (accession number: PRJEB48728).

### Cell viability assay

Cell viability was performed as described previously [37]. Briefly, cells were plated, at 10,000 cells per well, in growth medium on a 96-well black plate (Cat. #3603, Corning) for 24 h. After this, the chemotherapy drug was added to the well to its final concentration. Cells were cultured for a further 72 h, stained with DAPI by Hoechst 33,342 (Cat. #H3570, Life Technologies), and imaged in 20 fields for each well using an Evos FL Auto 2 imaging system (Cat. #AMAFD2000, Invitrogen). Images were counted using HCS studio cell analysis software V2.0 (Thermo Fisher Scientific, SX000041A, Waltham, MA, USA). Each data point was normalized to untreated control and generated in triplicate. Each experiment was run three times (*n* = 3). IC50 curves were generated in GraphPad Prism [(log (inhibitor) versus response (-variable slope four parameters)]. The Combination Index (CI) was calculated using ComboSyn software (ComboSyn Inc., http://www.combosyn.com/).

### Clonogenic assay

In brief, we performed the clonogenic assay using exponentially growing cells (70–80% confluence) seeded in 6-well plates (1000–2000 cells/well). Cells were treated with drugs for 3 days and then cultured without drugs for 7 to 10 days depending on the growth rate. Colonies were stained with 0.5% crystal violet (Cat. #61,135, Sigma Aldrich). Colonies of at least 50 cells were counted and the surviving fraction was normalized to the corresponding untreated control group.

### Luminescent caspase 3/7 activity assay

Luminescent caspase 3/7 activity assay was conducted with Caspase-Glo 3/7 Assay Systems (Cat. #G8091, Promega) as described by the manufacturer. The appropriate amounts of cells were seeded in white 96-well plates (Cat. #10,158,721, Thermo Fisher Scientific) and incubated for 24 h. Subsequently, drugs and DMSO were added to the cells and incubated for another 24 h. An equal amount of Caspase-3/7-Glo reagent was added to the wells; the contents of the wells were gently stirred and subsequently incubated for one hour at room temperature in the dark. Luminescence value was detected using a luminescent plate reader (Infinite 200 PRO, Tecan). Background signal (medium and Caspase-3/7-Glo reagent only) was subtracted and data were normalized to vehicle-treated cells.

### Western blot

Western blot analysis was performed as described [37]. In brief, cells were lysed in RIPA buffer (Cat. #89,900, Thermo Fisher Scientific) containing protease and phosphatase inhibitor (Cat. #78,442, Thermo Fisher Scientific), and the protein concentration was determined photometrically using a BCA Protein Assay Kit (Cat. #23,250, Thermo Fisher Scientific). Equal amounts of total protein were separated on 4–12% NUPAGE Bis–Tris gels (Cat. #NP0322BOX; Thermo Fisher Scientific) using the Mini Gel Tank chamber system (Cat. #A25977, Invitrogen), and proteins were transferred to a nitrocellulose membrane (Cat. #GE10600003, Sigma Aldrich). Membranes were then blocked in blocking buffer (Cat. # A0830.1000, AppliChem GmbH) for 1 h at room temperature and incubated with appropriate primary antibodies (Supplementary Table S3) overnight at 4 °C. HRP-linked anti-rabbit IgG or anti-mouse IgG were used as the secondary antibodies. Signal detection was performed using an Amersham Imager 600 (Pittsburgh, PA, USA) with SignalFire™ ECL Reagent (Cat. #6883S, Cell Signaling Technology).

### Cell cycle analysis and apoptosis assays

Treated cells were harvested and fixed with 75% ethanol at − 20 °C overnight, washed with ice-cold PBS, and incubated with RNase (200 µg/ml) for 60 min at 37 °C, and then stained with propidium iodide (PI; 50 µg/ml, Cat. #421,301, BioLegend). Quantitative analysis was performed with flow cytometry on a BD Biosciences LSRII flow cytometer.

For apoptosis assay, adherent cells treated with vehicle or drugs in the supernatant were detached with Trypsin–EDTA, collected, washed with PBS, and stained with FITC Annexin V Apoptosis Detection Kit I (Cat. #556,547, BD Pharmingen) according to the manufacturer's instructions. FITC-Annexin V uptake was measured on a BD Biosciences LSRII flow cytometer. The flow cytometry results were analyzed using FlowJo™ v10.8 Software (BD Life Sciences).

### In vivo mouse experiments

Experiments were performed on 8 to 10-week-old female C57BL/6JRj mice from Janvier Labs weighing 18–20 g. Experiments were compliant with the German legislation on the protection of animals and the Guide for the Care and Use of Laboratory Animals (Institute of Laboratory Animal Resources, National Research Council; NIH Guide, vol. 25, no. 28, 1996). Murine pancreatic ductal organoid culture was described previously [38]. In brief, TB32047 cells were harvested and evenly mixed with Matrigel (Cat. #354,230, VWR). Then, 50 μL domes of the cell/Matrigel suspension were plated into wells of a pre-warmed 24-well plate. After solidification of the Matrigel, 500 μL of pre-warmed feeding medium was added. The components of the feeding medium are listed in Supplementary Table S1. During culturing, medium was refreshed after 2 to 3 days. Seven days after seeding, organoids were harvested, and for each mouse, 50,000 organoids suspended in 20 µl of fresh Matrigel were orthotopically injected into the tail of the pancreas via laparotomy. The tumor-bearing mice, when tumor sizes reached 70–120 mm^3^, were randomized into 4 groups (5–8 mice/group), namely, vehicle (V, 10 μL/g 0.85% NaCl + DMSO, ip), THZ1 (T, 5 mg/kg, ip, 5 days/week), gemcitabine and nab-paclitaxel (GP, 50 mg/kg GEM ip, 5 mg/kg nab-PTX iv, Q4Dx4), and gemcitabine and nab-paclitaxel combined with THZ1 (GPT, 50 mg/kg GEM ip, 5 mg/kg nab-PTX iv, Q4Dx4, 5 mg/kg, THZ1 ip, 5 days/week). Tumor growth and progression were monitored daily, and tumor weight was estimated using the formula tumor weight (mg) = (length in mm) × (width in mm)^2^/2. Tumor-bearing mice (treatment, vehicle) were sacrificed on day 16 or when they became moribund before tumor volume reached 2000 mm^3^. At the end of each experiment, tumors were removed from the mice of all groups for further analysis.

### Statistical analysis

Unless otherwise indicated, statistical analyses were performed using GraphPad Prism (GraphPad Prism, RRID:SCR_002798; http://www.graphpad.com) version 8.0. The data in all experiments represent biological replicates (n) and are given as mean and standard deviations (mean ± SD), as described in the figure legends. The correlation analysis was performed using the Pearson correlation method. The unpaired, two-tailed Student’s *t* test, one-way ANOVA, or two-way ANOVA with a Tukey multiple comparison test were used to compare mean values, as shown in the figure legends. In all analyses, *p* < 0.05 was considered statistically significant.

## Results

### CRISPR-Cas9 screening identifies genes whose deficiency sensitizes pancreatic cancer cells to chemotherapy

**Fig. 1:**
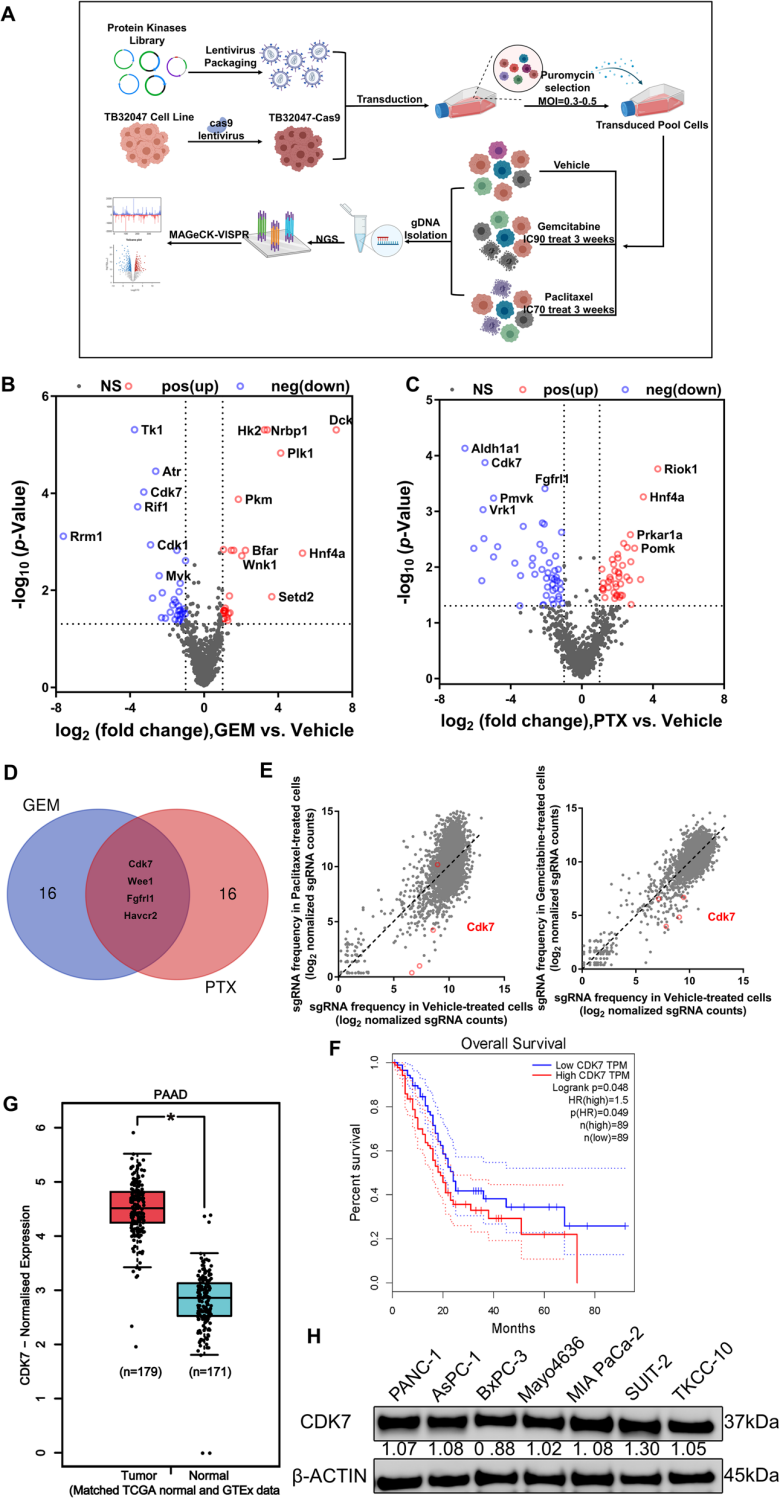
CRISPR-Cas9 knockout screens to identify chemosensitivity targets in pancreatic cancer cells treated with gemcitabine and paclitaxel. **A** Schematic diagram of the timeline and experimental procedure for CRISPR-Cas9 screening using protein kinase library. **B, C** Volcano plots showing log_2_-transformed sgRNA normalized fold change and -log_10_-transformed *p*-value of gemcitabine (**B**) and paclitaxel (**C**) versus vehicle-treated TB32047 cells, with significant genes in positive and negative selection highlighted in red and blue, respectively. **D** Venn diagram showing the shared genes of the top 20 candidate genes for gemcitabine and paclitaxel negative screening, which are *Cdk7, Wee1, Fgfrl1,* and *Havcr2.* **E** Scatterplots showing log_2_-transformed sgRNA normalized read counts of gemcitabine (left) and paclitaxel (right) versus vehicle-treated TB32047 cells, with sgRNAs targeting Cdk7 highlighted. **F** Data from TCGA-PDAC were applied for survival analysis. Kaplan–Meier survival analysis shows that higher CDK7 mRNA expression is associated with a tendency toward poor overall survival. **G** The analysis of CDK7 expression in PDAC tissues and normal pancreatic tissues was conducted using the TCGA (PDAC) and GTEx (Pancreas) databases. Taken together, the results indicate that CDK7 expression levels were significantly higher in pancreatic cancer than in the corresponding normal pancreatic tissue. **H** Western blot analysis revealed a variation in CDK7 expression between pancreatic cancer subtypes. * *p* < 0.05; *** *p* < 0.001, and **** *p* < 0.0001 were calculated using the unpaired Student’s *t* test

Poor response to chemotherapy accounts for the very low survival time and rate of patients with pancreatic cancer. Continuous treatment resulted in strong cell proliferation inhibition and cell death. This would also affect non-tumor cells and, importantly, hamper the evaluation of treatments concomitantly applied with chemotherapy. To explore new mediators of chemotherapy sensitivity, we performed CRISPR-Cas9 knockout screening in the pancreatic cancer cell line TB32047 derived from the KPC mouse model. We transduced TB32047-cas9 cells with a protein kinase library with additional sgRNAs enriched for PDAC-associated genes containing 3438 sgRNAs in total, which target 920 murine genes, and 110 non-targeting control sgRNAs. Transfected cells were selected by puromycin treatment, followed by continuous exposure to vehicle control or gemcitabine/paclitaxel chemotherapy for 21 days (Fig. 1A). We experimentally determined the optimal concentration of the drugs in TB32047 cells (Supplementary Fig. S1A and B). Continuous treatment of transfected TB32047 cells with gemcitabine at IC90 and paclitaxel at IC70 resulted in strong cell proliferation inhibition and cell death. Subsequently, genomic DNA was isolated from transfected and puromycin-selected cells were treated with vehicle control or gemcitabine or paclitaxel, and referred to as TB-C3W and TB-G3W/TB-P3W, respectively. sgRNAs were quantified via next-generation sequencing (Supplementary Fig. S1C, Supplementary Table S2).

We then analyzed the sgRNA counts of each group and scored genes targeted by sgRNA in TB-C3W versus TB-G3W/TB-P3W. We prioritized targets according to commonalities among sgRNAs, and genes with multiple sgRNAs showing a significant reduction in TB-C3W compared with TB-G3W/TB-P3W were considered for further analysis. We ranked individual sgRNAs based on log_2_ fold change (FC) <  − 1 and adjusted *p* < 0.05, and finally identified 33 genes as candidates in gemcitabine drug screening (Fig. 1B; Supplementary Table S4) and 47 genes in paclitaxel drug screening (Fig. 1C; Supplementary Table S4). Gene Ontology (GO) terms biological process analysis (DAVID Bioinformatics Resources 6.8) indicated that these genes were involved in cell division, protein phosphorylation, cell cycle regulation, mitotic nuclear division, DNA damage repair (DDR), apoptosis, and cellular response to DNA damage stimulus (Supplementary Fig. S1D and E).

We took the intersection of the top 20 genes in the two drug screenings with a total of four identical gene hits, namely, Cdk7, Wee1, Fgfrl1, and Havcr2 (Fig. 1D), whose loss of function via specific sgRNAs led to lethality in the presence of gemcitabine and paclitaxel (Supplementary Table S4). Interestingly, CDK7 is involved in a wide spectrum of biological processes, including cell cycle regulation and DNA damage response [39]. The majority of CDK7-targeting sgRNA standardized read counts were considerably lower following treatment with gemcitabine and paclitaxel compared to non-treated cells (Fig. 1E). Data from TCGA-PDAC indicated that increased CDK7 expression is statistically associated with poor overall survival in PDAC, and CDK7 is upregulated in pancreatic cancer compared to normal tissues (Fig. 1F and G). Furthermore, CDK7 is expressed in PC cell lines of different subtypes (Fig. 1H). For these reasons, we focused on analyzing the role of CDK7 in response to chemotherapy.

### Knockout of CDK7 combined with gemcitabine and paclitaxel chemotherapy enhances antiproliferative effects and apoptosis in pancreatic cancer cells

**Fig. 2:**
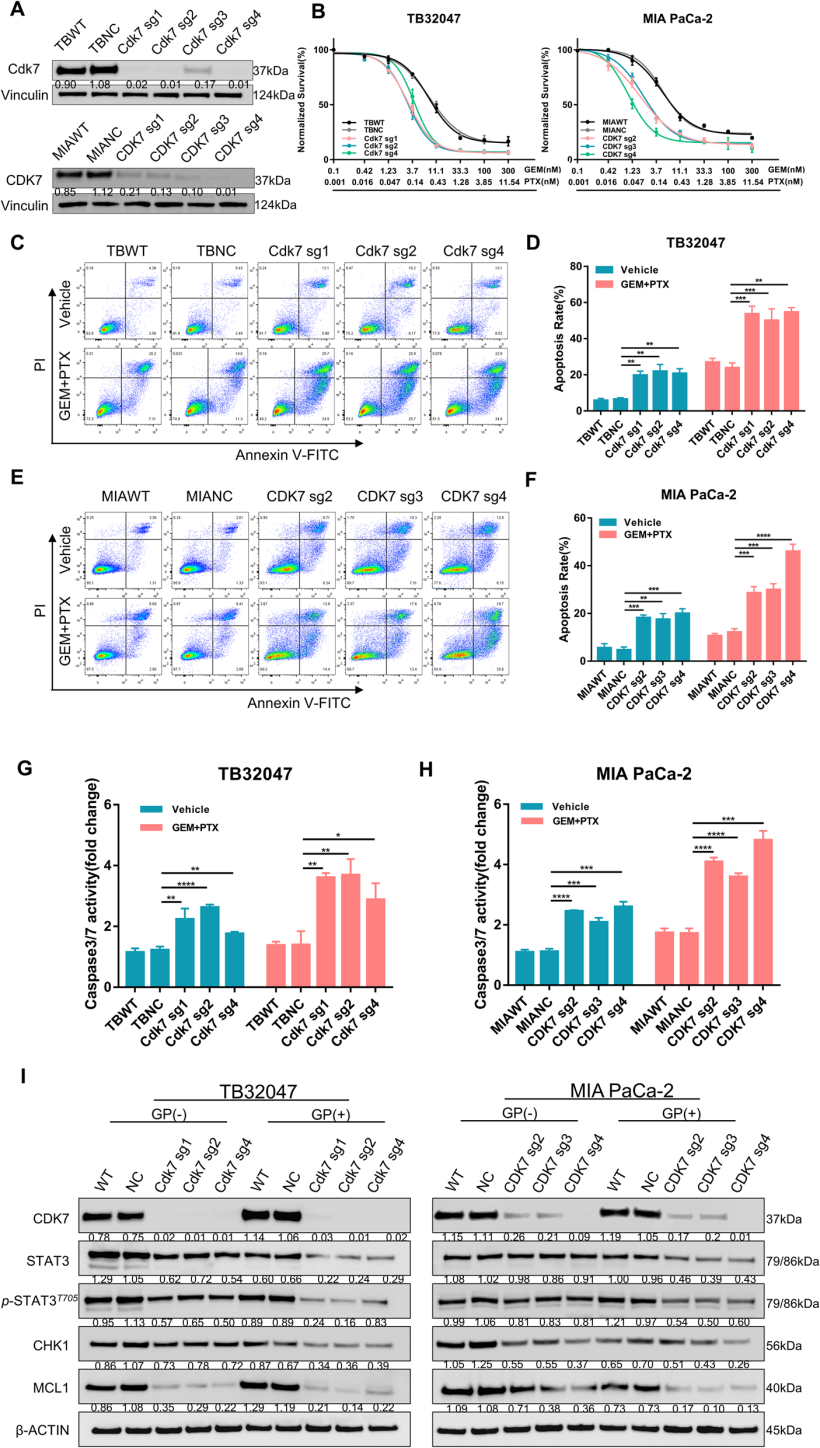
CDK7 knockout in combination with gemcitabine and paclitaxel treatment inhibits proliferation and increases apoptosis in pancreatic cancer cells. **A** Western blotting of TB32047 and MIA PaCa-2 cells transfected with CDK7 sgRNAs or non-targeting control. **B** The dose–response curves for GEM and PTX in TB32047 and MIA PaCa-2 CDK7 KO or NC cells. Seventy-two hours following treatment, cell survival was measured and normalized. Data are presented as the mean of three independent experiments (*n* = 3). **C–F** CDK7-WT (NC sgRNA) and CDK7-knockout TB32047 (**C**, **D**) cells, as well as MIA PaCa-2 (**E**, **F**) cells, were treated with vehicle or chemotherapy for 24 h and then analyzed for apoptosis using flow cytometry. Data are presented as the mean of three independent experiments (*n* = 3). ** *p* < 0.01; *** *p* < 0.001, and **** *p* < 0.0001 by two-way ANOVA with a Tukey multiple comparison test. **G** and **H** TB32047 (**G**) and MIA PaCa-2 (**H**) cells expressing CDK7 or control sgRNAs were treated with GEM and PTX; caspase 3/7 activity was measured 24 h later. Data are presented as mean ± SD (*n* = 3). * *p* < 0.05, ** *p* < 0.01, *** *p* < 0.001, and **** *p* < 0.0001 by two-way ANOVA with a Tukey multiple comparison test. **I**. Western blot analysis of TB32047 (left) and MIA PaCa-2 (right) cells transfected with non-targeting control (NC sgRNA) or CDK7-targeting sgRNAs (CDK7-sg1, CDK7-sg2, CDK7-sg3, CDK7-sg4) and treated for 24 h with vehicle or GEM plus PTX

Notwithstanding extensive existing work on the mechanism by which CDK7 regulates transcription and the cell cycle, no in-depth research has yet taken place on deletion of CDK7 to improve the performance of existing standard chemotherapy regimens in PDAC. To confirm CDK7 as a potential therapeutic target for enhancing the impact of chemotherapy, we generated CDK7-stable KO subclones by transfecting TB32047 and MIA PaCa-2 cells with four sgRNAs (Fig. 2A; Supplementary Table S4). Western blot of MIA PaCa-2 cells confirmed an efficient CDK7 knockout by sgRNA2, sgRNA3, and sgRNA4. These three subclones were used in the follow-up experiments (Fig. 2A). Dose-dependent viability assays and clonogenic assays indicated that CDK7-deficient cell lines were significantly more sensitive to both GEM and PTX than cells transduced with non-targeting control sgRNA (NC) (Fig. 2B; Supplementary Fig. S2A). Consistent with these observations, apoptosis by GEM and PTX were strongly upregulated in CDK7-deficient cells (Fig. 2C–F). Flow cytometry analysis revealed that this accounted for early and late apoptosis. Additionally, CDK7 knockout cell lines demonstrated higher caspase 3/7 activity (Fig. 2G and H). These findings suggest that CDK7 hampers GEM- and PTX-promoted apoptosis. Interestingly, our results show that CDK7 knockout also slightly increased cell apoptosis without chemotherapy (Fig. 2D and F); this may be related to the role of CDK7 in transcriptional control of DDR networks [39, 40].

Following this, we investigated the mechanism through which CDK7 deficiency results in chemosensitivity. CDK7 is required for RNAPII-mediated transcription and also functions as an integral component of the transcription factor TFIIH, which is involved in transcription initiation and DDR [32, 39]. We performed Western blot analysis of apoptosis and DDR-associated proteins in TB32047 and MIA PaCa-2 KO cell lines treated with vehicle or GEM and PTX. We observed substantial downregulation of phosphorylated STAT3 protein expression in the absence of CDK7, which was more pronounced in the presence of GEM and PTX (Fig. 2I). The phosphorylation level of STAT3 in CDK7 knockout cells decreased more markedly after chemotherapy. We also detected a decrease in the protein level of MCL1 after CDK7 deletion. Similarly, we determined the amount of expression of CHK1, an MCL1 regulatory target involved in cell cycle control and DNA damage repair. CHK1 consistently decreased in the presence of CDK7 knockout, and CHK1 was significantly decreased following 24 h of treatment. These findings would be compatible with CDK7 hampering activation of the STAT3-MCL1-CHK1 signaling cascade by chemotherapy (Fig. 6C).

### Targeted inhibition of CDK7 enhanced gemcitabine and paclitaxel chemotherapy response in pancreatic cancer in vitro

**Fig. 3:**
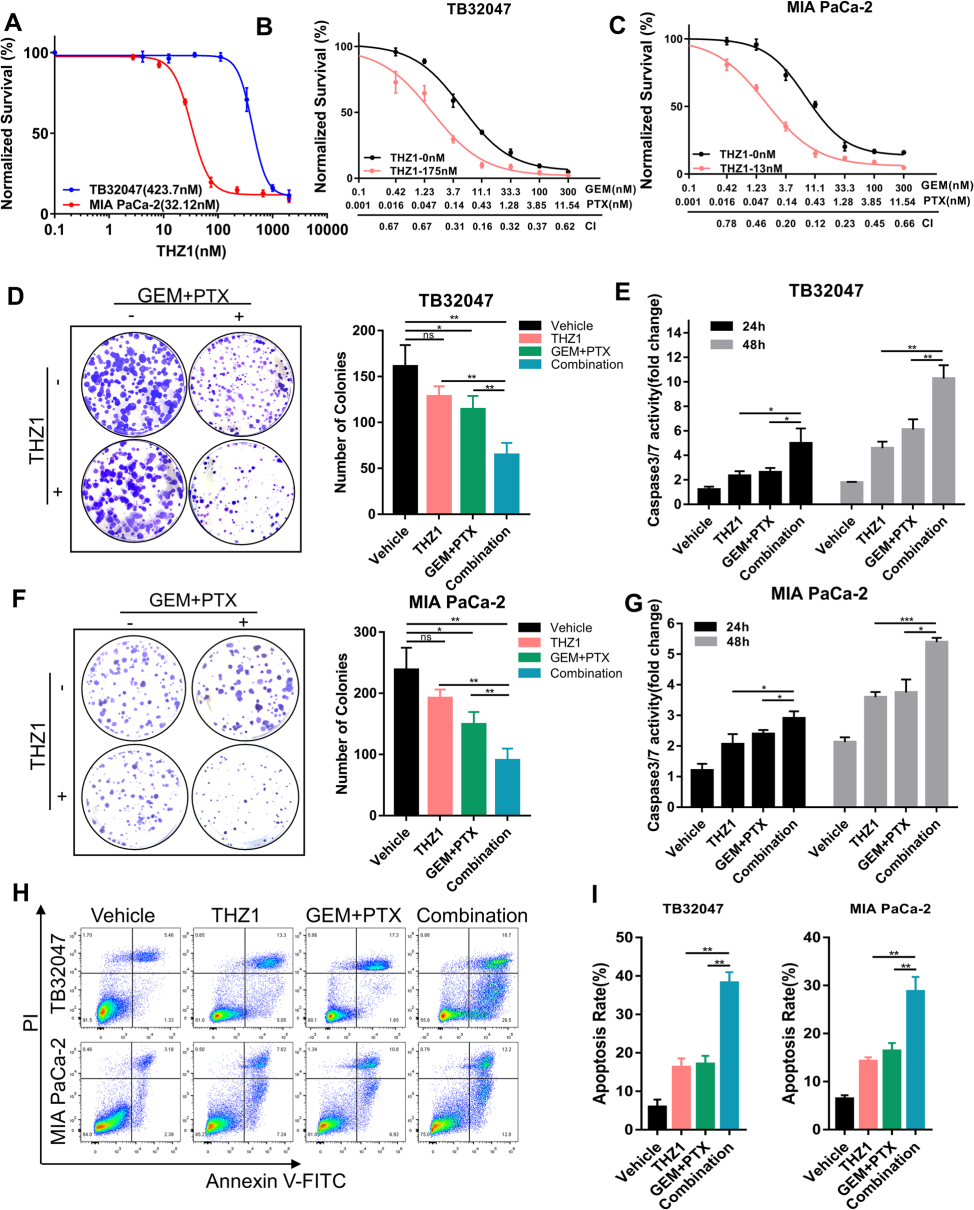
Targeted inhibition of CDK7 enhanced gemcitabine and paclitaxel chemotherapy response in pancreatic cancer in vitro. **A** Dose–response curves of TB32047 and MIA PaCa-2 wild-type cells to THZ1. Cell proliferation was evaluated 72 h after drug treatment. Data are presented as the mean of three independent experiments (n = 3). **B** and **C** The CDK7 inhibitor THZ1 enhances the antiproliferative impact of GEM and PTX in pancreatic cancer cells synergistically. Proliferation analysis was performed on TB32047 (**B**) and MIA PaCa-2 (**C**) cells treated for 72 h with THZ1 and GEM/PTX alone or in combination. The plot of fraction affected versus combination index is shown below the x-axis. CI < 1.0, synergism. **D** and **F** Clonogenic assay of TB32047 (**D**) and MIA PaCa-2 (**F**) cells treated with vehicle or drugs for 72 h and cultured without drugs for an additional 7–10 days. The cells remaining after the treatment were fixed and stained with crystal violet. Representative images (left) and quantifications (right) of three independent experiments (*n* = 3) are shown. * *p* < 0.05 and ** *p* < 0.01 by one-way ANOVA with a Tukey multiple comparison test. ns: not significant. **E** and **G**. Caspase 3/7 activity was evaluated in TB32047 (**E**) and MIA PaCa-2 (**G**) cells treated for 72 h with vehicle or drugs. Data are presented as mean ± SD (n = 3). * *p* < 0.05, ** *p* < 0.01, and *** *p* < 0.001 by two-way ANOVA with a Tukey multiple comparison test. **H** and **I** Flow cytometry-based apoptotic assay of TB32047 and MIA PaCa-2 cells treated for 72 h with drugs or drug combination for 72 h. Representative images (**H**) and quantification (**I**) of three independent experiments (*n* = 3) are shown. ** *p* < 0.005 by two-way ANOVA with a Tukey multiple comparison test

The experiments described above confirm that CDK7 knockout sensitizes PDAC to GEM/PTX therapy by increasing antiproliferative effects and apoptosis. We next explored whether the CDK7-targeted inhibitor THZ1 may have the same synergistic effect in vitro. Dose–response curves performed on a panel of human PDAC cell lines (MIA PaCa-2, AsPC-1, SUIT-2, BxPC-3, Mayo4636, TKCC-10) and TB32047 indicated that THZ1 inhibited PDAC cell viability in a dose-dependent manner, with the IC50 ranging from 26.08 nM to 423.7 nM (Fig. 3A; Supplementary Fig. S2B).

To analyze whether CDK7 inhibitor could work synergistically with GEM and PTX in suppressing PDAC growth, we used THZ1 at a dose inhibiting proliferation by less than 20% (IC20) in the PDAC cell lines given above, in conjunction with a concentration gradient of GEM and PTX, for 72 h. The results indicate that GEM and PTX reduced PDAC cell viability in a dose-dependent manner, while the combination of THZ1 with GEM/PTX generated a substantial synergistic effect (CI < 1), resulting in a more marked reduction in cell viability than the GEM/PTX regimen (Fig. 3B and C; Supplementary Fig. S2C-G). Clonogenic assays revealed that the combination treatment group had considerably fewer colonies than groups treated with THZ1 or GEM/PTX alone (Fig. 3D and F). According to the caspase 3/7 activity assay, the combined treatment group exhibited significantly higher caspase 3/7 activity than either chemotherapy or THZ1 alone, suggesting that the cytotoxicity generated by combination treatment was more effective (Fig. 3E and G). Furthermore, flow cytometry analysis confirmed that the combination treatment was superior to single agents, as manifested by the more pronounced activation of apoptotic cell death induced by combined THZ1 and GEM/PTX (Fig. 3H and I; Supplementary Fig. S2H). In conclusion, these results confirmed that the targeted inhibition of CDK7 amplifies the toxicity of chemotherapy, rendering PDAC cells more vulnerable to GEM/PTX chemotherapy.

### CDK7 inhibition combined with standard chemotherapy suppresses PDAC tumor growth in vivo

The results outlined above indicate that knockout or suppression of CDK7 effectively improved the response to GEM and PTX in PDAC. We proceeded with evaluating the impact of combined CDK7 inhibition and chemotherapy in vivo. To establish an orthotopic mouse model of pancreatic cancer, we used TB32047 organoids. Tumor-bearing mice were treated with GEM (50 mg/kg) and nab-PTX (5 mg/kg) once every 4 days and THZ1 (5 mg/kg) 5 consecutive days a week. Notably, the doses of chemotherapy and THZ1 used in our study were below clinically achievable concentrations. As a consequence, none of the combinations caused substantial weight loss in mice, and no additional toxicity was observed during the treatment (Supplementary Fig. S2I).

**Fig. 4:**
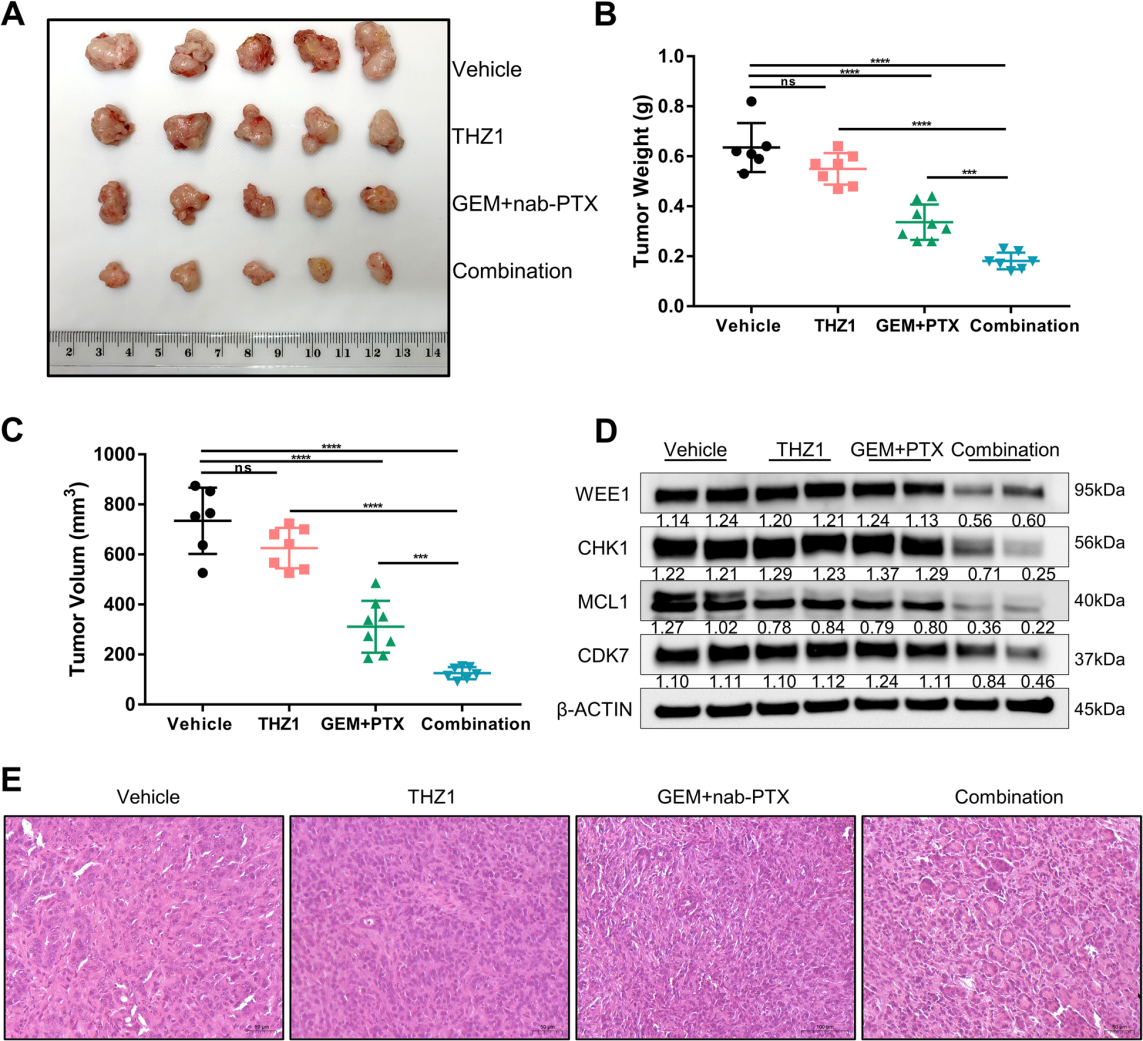
Combined CDK7 inhibition with standard chemotherapy suppresses PDAC tumor growth in vivo. **A** Images of TB32047 orthotopic mouse model of pancreatic cancer after treatment for 16 days. **B** and **C** Relative tumor weights (**B**) and volumes (**C**) of TB32047 orthotopic pancreatic cancer mouse model after treatment. *** *p* < 0.001 and **** *p* < 0.0001 by one-way ANOVA with a Tukey multiple comparison test. ns: not significant. **D** Western blot analysis of tissue samples from mice with pancreatic cancer that had been treated with vehicle, GEM/nab-PTX, or a combination. **E** Hematoxylin and eosin (H&E) staining demonstrated that the combination of THZ1 and GEM with nab-PTX decreased tumor cell content compared to the group receiving single components and the control group. Scale bar (black), 50 μm

Treatment with GEM and nab-PTX slightly reduced tumor volume, while THZ1 alone had no effect on tumor volume. The combination treatment considerably increased the effectiveness of chemotherapy in an orthotopic pancreatic cancer mouse model by significantly decreasing tumor size (Fig. 4A), volume, and weight (Fig. 4B and C). The combination of CDK7 inhibitor and GEM + nab-PTX therefore exhibited anti-tumorigenic activity which was superior to chemotherapy alone. The high synergistic impact of THZ1 was consistent with our in vitro results (Fig. 2 and Fig. 3). We performed Western blotting to evaluate the expression levels of cell cycle- and apoptosis-related proteins in tumor samples from different treatment groups. Combination treatment for PDAC tumors resulted in lower WEE1 and CHK1 expression than monotherapy. The expression of MCL1 in PDAC tumors was reduced following the combination of treatments, suggesting an increase in apoptosis (Fig. 4D). Based on evaluation of tumor slides, we also observed a decrease in tumor cell content in the tumors analyzed (Fig. 4E). Briefly summarized, our in vivo results validate the effectiveness of combined CDK7 inhibition as a strategy for boosting the efficacy of standardized treatment and overcoming chemoresistance in PDAC.

### CDK7 inhibition induces cell cycle arrest, apoptotic cell death, and DNA damage through the STAT3-MCL1-CHK1 axis

**Fig. 5:**
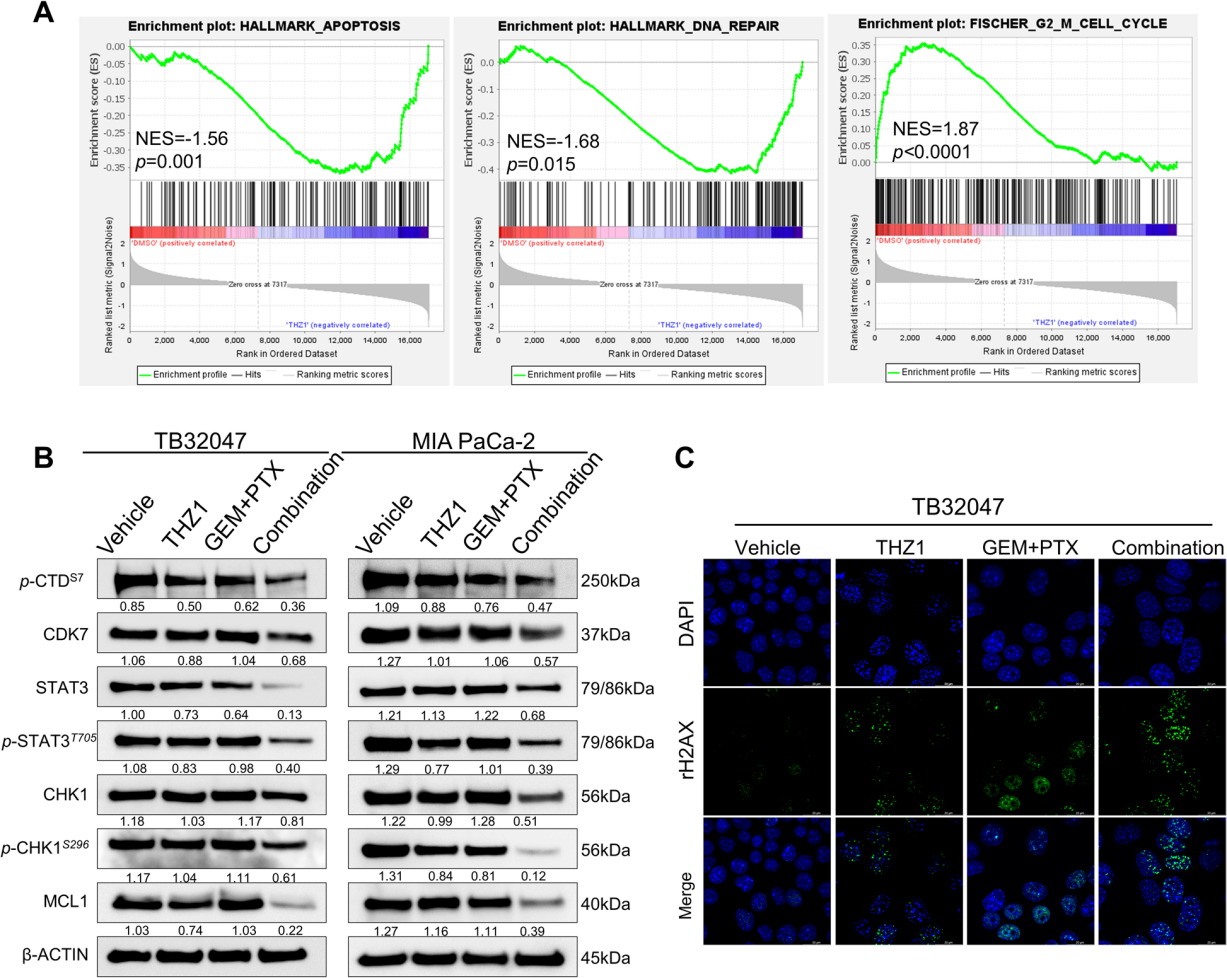
CDK7 inhibition induces cell cycle arrest, apoptotic cell death and DNA damage through the STAT3-MCL1-CHK1 axis. **A** Gene set enrichment analysis (GSEA) on the RNA-sequencing data of BXPC-3, Mia PaCa-2, and PANC-1 cells treated with THZ1, a selective CDK7 inhibitor. Transcriptomic gene expression data are based on the GEO dataset GSE121273. **B** Western blotting of TB32047 and MIA PaCa-2 cells treated with vehicle (DMSO), THZ1, and GEM plus PTX, alone or in combination, for 24 h. **C** Immunofluorescence of TB32047 cells treated with vehicle (DMSO), THZ1, and GEM/PTX, alone or in combination, for 24 h. Cells were subsequently stained with antibodies to γH2AX (green) and DAPI (blue; nuclei). Scale bar (white), 20 μm

We attempted to further explore the role of CDK7 in signaling pathway regulation and in mediating chemoresistance in PDAC. To investigate the potential molecular mechanism of chemotherapy and CDK7 inhibition in combination, we first interrogated the Gene Expression Omnibus (GEO) datasets and performed gene set enrichment analysis (GSEA) on the RNA-sequencing data of BXPC-3, MIA PaCa-2, and PANC-1 cells treated with THZ1 [32]. Previous studies have demonstrated that CDK7 inhibition significantly decreases gene transcription, with a preference for mitotic cell cycle and NF-κB signaling-related transcripts [32]. We found, via further analysis of transcriptional data, that THZ1 substantially decreased the gene features of DDR-related genes, including NHEJ1, CHK1, CHK2, ATM, ATR, RAD51, RAD51B, RAD51D, etc.; G2-M checkpoint-related genes, including WEE1, E2F1, CDC25A, CDC25B, CDC25C, CCNB1, CDK1, CDK2, etc.; and apoptosis-related genes, including BAX, BCL2, BID, CASP3, CASP7, CASP9, CDNK1A, MCL1, etc. (Fig. 5A). Notably, CDK7 is also strongly positively correlated with the expression of key genes involved in cell cycle and DDR; we found this via the analysis of TCGA transcriptomic data from 185 PDAC patients, in particular, CDC25C, CCNB1, CDC25B, CDK1, NHEJ1, CHK1 and RAD51, with Pearson's correlation coefficients being 0.42, 0.51, 0.31, 0.41, 0.48, 0.24, and 0.24, respectively (Supplementary Fig. S3A).

To further demonstrate that targeted inhibition of CDK7 results in a decrease in global transcript levels and downregulation of the above-mentioned protein levels, we treated TB32047 and MIA PaCa-2 cells with THZ1 in a concentration gradient and a time gradient to ascertain the effects of CDK7 inhibition on cell cycle regulation and apoptosis. Importantly, p-CTD^*Ser7*^ progressively decreased in a time- and dose-dependent manner, a finding related to the role of CDK7 in RNAPII-mediated transcription. Ser7 of the C-terminal domain (CTD) heptapeptide repeat is phosphorylated during the active transcription cycle, which is required for efficient transcription of small nuclear RNA genes [41]. Furthermore, WEE1, phosphorylated WEE1, phosphorylated CDK1, and phosphorylated CDK2 all decreased dose- and time-dependently. Simultaneously, when the dose or time period of exposure was increased, the cleaved PARP was upregulated, indicating that THZ1 also regulated apoptosis in a time- and dose-dependent manner (Supplementary Fig. S3B and C). Cell cycle analyses revealed increased G2-M arrest in a dose-dependent pattern in the two aforementioned cell lines (Supplementary Fig. S3D and E).

Having established that deletion of CDK7 results in cell cycle arrest and increased apoptosis through the STAT3-MCL1-CHK1 pathway, rendering PDAC susceptible to GEM/PTX therapy, we became interested in whether the same mechanism improves the efficacy of chemotherapy. We treated TB32047 and MIA PaCa-2 cells for 24 h with THZ1 at a dosage that has no impact on cell proliferation in conjunction with chemotherapy. Consistent with the impact of CDK7 deletion, CDK7 inhibition under chemotherapy led to a decrease in STAT3/pSTAT3 that was not observed by monotherapeutic treatments. Cell cycle checkpoint protein CHK1 and its Ser296 phosphorylation site were also considerably suppressed, indicating an increase in the severity of cell cycle arrest and DNA damage. The level of the anti-apoptotic protein MCL1 was also considerably decreased, demonstrating that apoptosis was induced, which was consistent with the apoptosis experiment results (Fig. 5B). Cell cycle analysis revealed that G2-M arrest of TB32047 and MIA PaCa-2 was more significant in inhibitors combined with chemotherapies than in single-drug treatment (Supplementary Figure S3F and S3G). Immunofluorescence experiments confirmed that the combined treatment significantly increased the number of γH2AX foci compared to the THZ1 or GEM/PTX treatment group (Fig. 5C), indicating that the combined treatment might result in the highest percentage of DNA double-strand breaks (DSB) causing cell death.

### Targeted inhibition of CDK7 reversed chemoresistance in pancreatic cancer cells

**Fig. 6:**
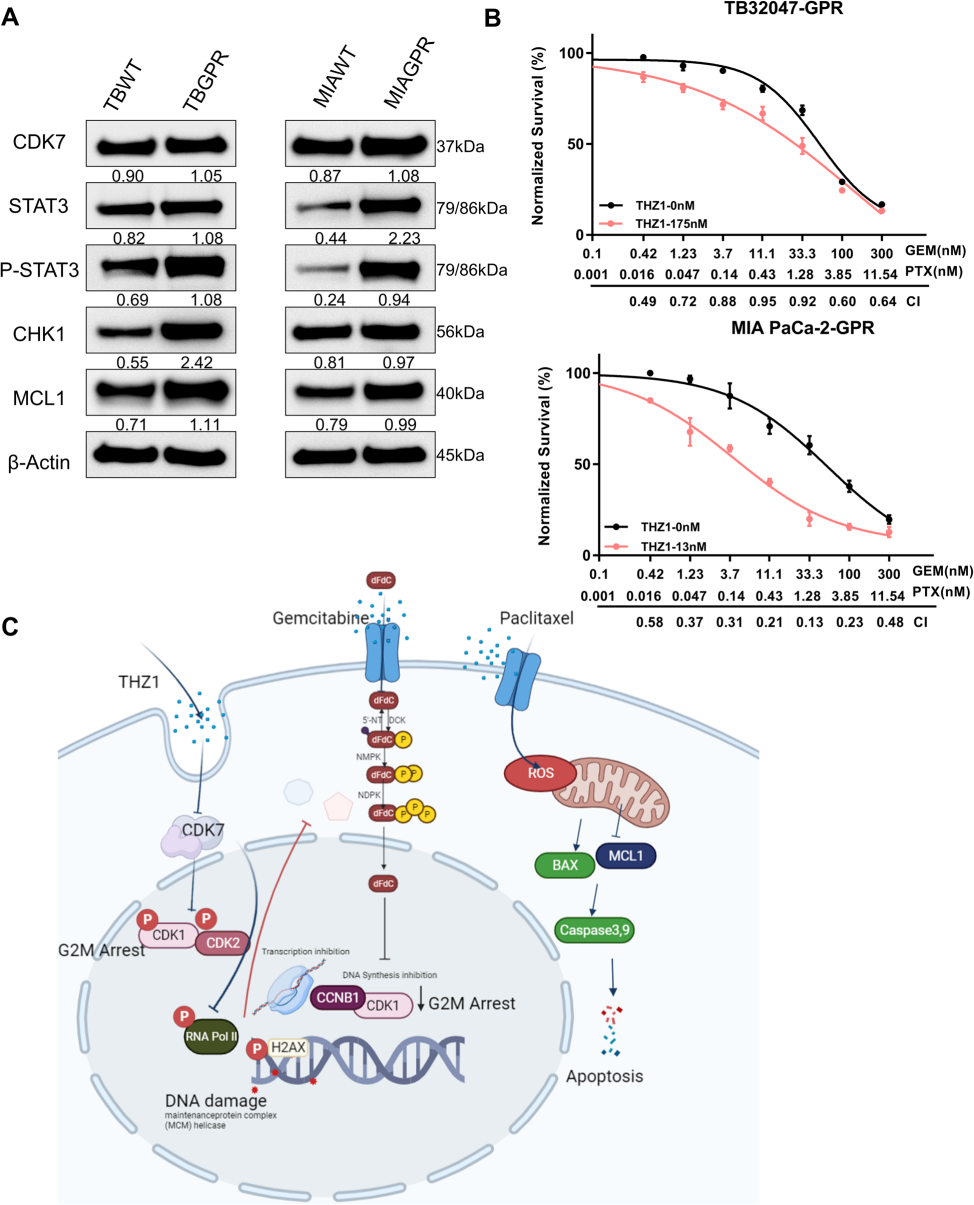
Targeted inhibition of CDK7 reverses chemoresistance in pancreatic cancer. **A** Western blotting of TB32047 and MIA PaCa-2 gemcitabine- and paclitaxel-resistant cells (TBGPR, MIAR) and parental cells (TBWT, MIAWT). **B** The CDK7 inhibitor THZ1 synergistically enhances the antiproliferative effects of GEM and PTX in TBGPR and MIAGPR cells. The proliferation was determined after 72 h of treatment with THZ1 and GEM/PTX alone or in combination. Below is a plot of the fraction impacted versus the combination index. CI < 1.0, synergism. **C** Diagrammatic representation of the research outcome. Gemcitabine and paclitaxel combined with CDK7 inhibition induces DNA damage, G2-M arrest, and subsequent apoptosis

Our findings suggest that inhibiting CDK7 enhances the efficacy of standard chemotherapy in generating cell cycle arrest, DNA damage, and ultimately apoptosis in PDAC. In a final set of experiments, we wanted to explore whether chemoresistance can be reversed by THZ1. Acquired resistance (TBGPR and MIAGPR) was achieved by chronically treating TB32047 and MIA PaCa-2 cells with incremental doses of GEM and PTX, which resulted in 5–tenfold increases in IC50 in chemoresistant cells compared to parental cells (Fig. 3B and C; Fig. 6B).

Interestingly, CDK7, STAT3, P-STAT3^*Tyr705*^, CHK1, and MCL1 signal activation was clearly increased in chemoresistant cells compared to parental cells (Fig. 6A). Notably, THZ1 also restored GEM/PTX sensitivity in chemoresistant TBGPR and MIAGPR cells, as revealed by the cell viability assay. The cell viability assay revealed a high synergistic effect between THZ1 and GEM/PTX in the two resistant cell lines (Fig. 6B). Surprisingly, chemoresistant cell lines demonstrated increased susceptibility to THZ1 (Supplementary Fig. S4A and B), which may be due to the persistently high CDK7 expression in TBGPR and MIAGPR. Similarly, flow cytometry analysis in TBGPR cell lines showed that the combined THZ1 and GEM/PTX treatment induced higher levels of apoptosis than the single treatment regimen (Fig. S4C). These results indicate that CDK7 contributes to chemoresistance and that THZ1 overrides this process, rendering refractory PDAC cells susceptible to GEM/PTX chemotherapy (Fig. 6C). Despite these optimistic findings, we have only touched the surface of the causes of chemoresistance in PDAC; the situation remains significantly more complex, and the few molecules taken into account here cannot unravel it fully.

## Discussion

Distinct from some other cancer types, the success of targeted therapies in PDAC remains disappointingly low with respect to disease-free and overall survival [2, 7, 42]. This includes chemotherapy and is at least in part due to chemoresistance. Thus, chemosensitizers have come into focus [7]. Here, we used a sgRNA library focused on kinases and PDAC-associated genes and identified CDK7 as a candidate for chemotherapy sensitization in pancreatic cancer by adopting a systematic approach of negative selection of CRISPR-Cas9. We have demonstrated that the efficient inhibition of CDK7 improved cell apoptosis and DNA damage after chemotherapy, a mechanism that works, at least in part, by decreasing the signal transduction of the STAT3-MCL1-CHK1 pathway, so as to promote the impact of GEM and PTX. THZ1, a covalent inhibitor of CDK7, has shown promising therapeutic effects in preclinical models for a range of malignancies, including high-grade glioma [43, 44], hepatocellular carcinoma [45, 46], and non-small cell lung cancer [28]. We here examined the therapeutic impact of CDK7 inhibition in a preclinical PDAC tumor model and discovered that targeting CDK7 provides a new option to enhance the efficacy of chemotherapy. Moreover, there exists a link between chemoresistance and the upregulation of STAT signal-dependent gene transcription, whereby chemoresistant PDAC becomes particularly sensitive to THZ1. Combined therapy successfully induced chemo-sensibilization, pointing to a novel approach to overcoming chemoresistance in pancreatic cancer [47]. In summary, our results provide a mechanistic basis for the combination of CDK7 inhibitors and standard chemotherapy in the treatment of pancreatic cancer, and support the application of CRISPR-Cas9 functional genomics in the identification of novel therapeutic targets and the development of new targeted drugs to increase the efficacy of chemotherapies.

Apart from its normal involvement in the cell cycle, CDK7 is essential for RNA transcription mediated by RNA polymerase II [28]. Inhibiting CDK7 specifically reduced the activity of STAT3 and other cell death-related proteins. STAT3 mediates the expression of multiple genes in response to cellular stimuli and therefore plays a key role in many cellular processes, such as cell growth and apoptosis [48, 49]. Weakening the STAT pathway enhanced the susceptibility of surviving cells to GEM and PTX treatment. Even in cells with a STAT3 mutation, the STAT signaling system is particularly vulnerable to THZ1; STAT3 chromatin binding and highly transcribed target genes such MYC, PIM1, MCL1, CD30, and IL2RA are reduced in mutant cells where CDK7 is inhibited [48–50]. MCL1 and BCL-XL expression in tumor cells is decreased by THZ1, making tumor cells more responsive to BH3 mimics [48]. Therefore, the combination of THZ1 and BH3 mimics is also a potential option for targeted therapy.

Cancer cells escape apoptosis by a variety of mechanisms, including increased production of pro-survival proteins (BCL2, BCLXL, or MCL1) [51]. The BCL2 protein family is critical to tumor cell survival [51, 52], and PDAC, in particular, is strongly dependent on the anti-apoptotic protein MCL1 [52, 53]. Our data demonstrated that CDK7 deletion dramatically reduced the amount of MCL1 protein, an impact enhanced when coupled with GEM/PTX therapy. Thus, when chemotherapy was combined with THZ1 at a dosage that did not affect proliferation, MCL1 was also considerably reduced, indicating that THZ1 may combine effectively with chemotherapy to induce tumor cell death synergistically. In addition, our experimental data confirm the function of CDK7 in cell cycle control. Inhibition of CDK7 decreased the phosphorylation of G2-M cell cycle-related proteins WEE1, CDK1, and CDK2, resulting in an incremental arrest of the G2-M phase by THZ1 and a reduction in cell proliferation. The serine-threonine kinase WEE1 modulates G2-M checkpoint transition [54–56]. WEE1 induces G2-M arrest by inhibiting CDK1 (Cdc2) phosphorylation on Tyr15, preventing entry into mitosis and allowing DNA repair during DNA damage [54]. WEE1 inhibition may induce high CDK1 activity and cause the cell to proceed through the G2-M checkpoint without properly repairing DNA damage, resulting in mitotic catastrophe and cell death [55]. Furthermore, WEE1 inhibition catalyzes CDK2 phosphorylation and increases its activity, resulting in aberrant DNA replication and DSBs [54–56]. CDK1 is essential for mammalian cell proliferation and is the only CDK that can initiate the onset of mitosis. DNA replication and CDK1 activity are mediated by checkpoint signaling kinases such as CHK1 and WEE1, which suppress the activity of CDK1 via inhibitory phosphorylation [57]. Moreover, perturbations in chromosomal stability and aspects of S phase and G2-M control mediated by CDK2 and CDK1 are pivotal tumorigenic events [57, 58]. Interestingly, previous work has shown that the inhibition of CDK7 may reduce CDK1 and CDK2 activity, which would lead to the degradation of MCL1 [59].

In addition to its typical role in the cell cycle, CDK7 is involved in DDR [39]. As a transcriptional activator, the tumor suppressor protein p53 regulates the cell response to DNA damage by inducing apoptosis and cell cycle arrest [39]. p53 and the CDK-activating complex (CAK) share a high degree of commonality in terms of cell cycle and transcriptional control, and CAK phosphorylates p53 at the Ser33 site, a regulatory site of DDR [39, 44]. During nucleotide excision repair (NER), the CAK is released from the core TFIIH, which promotes the incision/excision of the damaged oligonucleotide and thereby the repair of the DNA [60]. In view of these facts, we concentrated on the relationship between CDK7-mediated transcriptional regulation and DNA damage. Previous studies have shown that CDK7 inhibition alters RNA Pol II and MYC association at DNA repair genes. Blocking CDK7 activity sensitizes cells to DNA damage, leading to accrual of γH2AX foci [39], a marker of early DSB [61]. Combining THZ1 with chemotherapy enhanced γH2AX foci and reduced CHK1 phosphorylation, thereby increasing radiation-induced apoptotic signaling. As an overall result, G2-M cell cycle arrest, DNA damage, and apoptosis strongly promote the efficacy of chemotherapy. These findings provide evidence for a causal connection between DNA damage and checkpoint mechanisms generated by the combined use of THZ1 and GEM/PTX.

We performed protein kinase library screening by applying chemotherapeutic stress in the KPC mouse model-derived cell line TB32047. Recent research has revealed that even among pancreatic cancer cell lines, genetic and behavioral features between cell lines of different origins and subtypes were quite different. In particular, pancreatic cancer subtypes are critically associated with treatment response during DNA damage response and replication stress [62, 63]. Therefore, we suggest that crossover or combination screens between different cell lines and different species should be continued at least with caution in subsequent studies, thus seeking key targets to overcome chemoresistance. In the present study, through a series of in vivo and in vitro experiments, we confirmed that CDK7 is involved in chemoresistance of GEM/PTX at least partially through the STAT3-MCL1-CHK1 signaling pathway. However, further studies are still needed to elucidate the regulatory relationship between CDK7 and the STAT3-MCL1-CHK1 axis and GEM/PTX chemoresistance, and to further develop combinations of drugs such as STAT3 and CHK1 inhibitors to overcome chemoresistance in PDAC.

## Conclusions

We demonstrated here that CDK7 is a novel therapeutic target for pancreatic cancer, which relies on its promotion of cell cycle progression, as well as its interference with DNA damage/DNA damage repair and apoptosis induction. CDK7 inhibition and GEM/PTX act synergistically such that the therapeutic efficacy of the combined application exceeds that of the individual drugs without any adverse effect, at least in a preclinical mouse model, as compared to monotherapy. We are therefore able to report a possible novel combination treatment regimen for overcoming PDAC chemoresistance. The efficacy of the regimen underscores the unique complementarities of CDK7 inhibition with standard chemotherapy and urges clinical trials in patients with PDAC and possibly other malignancies.

## Supplementary Information

**Additional file 1:** **Figure S1.** A kinome-wide CRISPR screen in pancreatic cancer cells. **Figure S2.** Targeted inhibition of CDK7 enhances gemcitabine and paclitaxel chemotherapy response in pancreatic cancer in vitro and in vivo. **Figure S3.** CDK7 inhibition induces cell cycle arrest, apoptotic cell death, and DNA damage. **Figure S4.** Targeted inhibition of CDK7 reverses chemoresistance in pancreatic cancer. **Table S1.** Cell lines. **Table S2.** Plasmids and primers list. **Table S3.** Antibody list. **Table S4.** List of CRISPR-Cas9 screening results of gemcitabine and paclitaxel in TB32047 cells.

## Data Availability

Raw data of the CRISPR/Cas9 screening data are available in the EBI database; accession number: PRJEB48728.

## Abbreviations

PDAC: Pancreatic ductal adenocarcinoma
PC: Pancreatic cancer
CDK7: Cyclin-dependent kinase 7
CAK: CDK-activating complex
CTD: C-terminal domain
KO: Knockout
ATCC: American Type Culture Collection
PDCLS: Patient-derived cell lines
CSHL: Cold Spring Harbor Laboratory
GEO: Gene Expression Omnibus
GSEA: Gene set enrichment analysis
DDR: DNA damage repair
DSB: Double-strand break
