# Supplementary material for: CDK7 inhibition augments response to multidrug chemotherapy in pancreatic cancer
Source: J Exp Clin Cancer Res. 2022 Aug 10;41:241. doi: 10.1186/s13046-022-02443-w (PMC9364549; doi:10.1186/s13046-022-02443-w)
Supplement: Supplementary file 1 — Additional file 1: Figure S1. A kinome-wide CRISPR screen in pancreatic cancer cells. Figure S2. Targeted inhibition of CDK7 enhances gemcitabine and paclitaxel chemotherapy response in pancreatic cancer in vitro and in vivo. Figure S3. CDK7 inhibition induces cell cycle arrest, apoptotic cell death, and DNA damage. Figure S4. Targeted inhibition of CDK7 reverses chemoresistance in pancreatic cancer. Table S1. Cell lines. Table S2. Plasmids and primers list. Table S3. Antibody list. Table S4. List of CRISPR-Cas9 screening results of gemcitabine and paclitaxel in TB32047 cells. [file 13046_2022_2443_MOESM1_ESM.docx]

**Supplementary Information for**

**CDK7 inhibition augments the response against multidrug chemotherapy in pancreatic cancer**

Siyuan Zeng,^1*^ Bin Lan,^1*^ Xiaofan Ren,^1^ Shuman Zhang,^1^ **Daniel Schreyer,^2^** Markus Eckstein,^3^ Hai Yang,^1^ Nathalie Britzen-Laurent,^1^ Andreas Dahl,^4^ Debabrata Mukhopadhyay,^5^ David Chang,^6,7^ Isabella Kutschick,^1^ Susanne Pfeffer,^1^ Peter Bailey,^2^ Andrew Biankin,^6,7^ Robert Grützmann,^1+^ Christian Pilarsky.^1+#^

#To whom correspondence may be addressed. Email: [Christian.Pilarsky@uk-erlangen.de](mailto:Christian.Pilarsky@uk-erlangen.de)

**Supplementary Methods**

**Public database mining, correlation, prognostic, and gene set enrichment analysis (GSEA)**

The TCGA public databases were utilized to analyze CDK7 expression, correlation, and prognosis in PDAC. TCGA PDAC normalized counts were downloaded using the TCGAbiolinks R package. Gene correlation analysis was performed using Pearson correlation. Survival analysis was performed using the Kaplan–Meier method and the difference was tested with the log-rank test. Transcriptomic data from BxPC-3, PANC-1, and MIA PaCa-2 cells treated with THZ1 (GSE121273) [[32](#_ENREF_32)] were obtained from the GEO database and analyzed using GSEA software. A *p*-value < 0.05 was considered statistically significant.

**CRISPR-Cas9 gene editing**

CDK7 was knocked out by the CRISPR-Cas9 gene editing system in TB32047 and MIA PaCa-2 cell lines, as previously described [[37](#_ENREF_37)]. The sgRNAs targeting CDK7 were synthesized by Eurofins Genomics and are listed in Supplementary Table S2. Cloning was performed using pSpCas9(BB)-2A-Puro (PX459) V2.0 vector, which was a gift from Feng Zhang (Addgene, RRID: Addgene_48139) [[36](#_ENREF_36)]. The ligated vector was inserted into Endura Electrocompetent Cells (Cat. #60242-1, Lucigen). The above-mentioned cell lines were transfected with CDK7 knockout plasmid by lipofectamine transfection reagent for 24 h; after this, the transfected cells were selected with puromycin (concentration 10 ng/μL) for three days. After growth, Western blotting was performed to detect the knockout.

**Immunofluorescence and H&E stain**

For immunofluorescence, cells were cultured on poly-lysine-treated cover slides, fixed with 4% paraformaldehyde for 15 min at room temperature (RT) and permeabilized with 0.1% Triton X-100/PBS at RT for 15 min. Fixed cells were then blocked with 10% goat serum and incubated with primary antibodies overnight at 4°C and 1 h at RT with Alexa Fluor 488 goat anti-Rabbit IgG (Cat. #A11034, Invitrogen). Nuclei were counterstained by DAPI Solution (Cat. #62248, Life Technologies). Images were acquired on a Leica LSM microscope (Leica Microsystems CMS GmbH, Wetzlar, Germany) and processed using Leica Application Suite X software.

For H&E staining, tissue specimens were fixed in 10% buffered formalin and paraffin-embedded for further histological work up. Four micrometers thick tissue sections were used to prepare staining with hematoxylin and eosin (H&E). Stainings were then scanned using the P250 slide scanner (3DHISTECH Ltd, Hungary) and visualized in SlideViewer v.2.5 (3DHISTECH Ltd, Hungary).

**Supplementary Figures**

**Figure S1.**

**
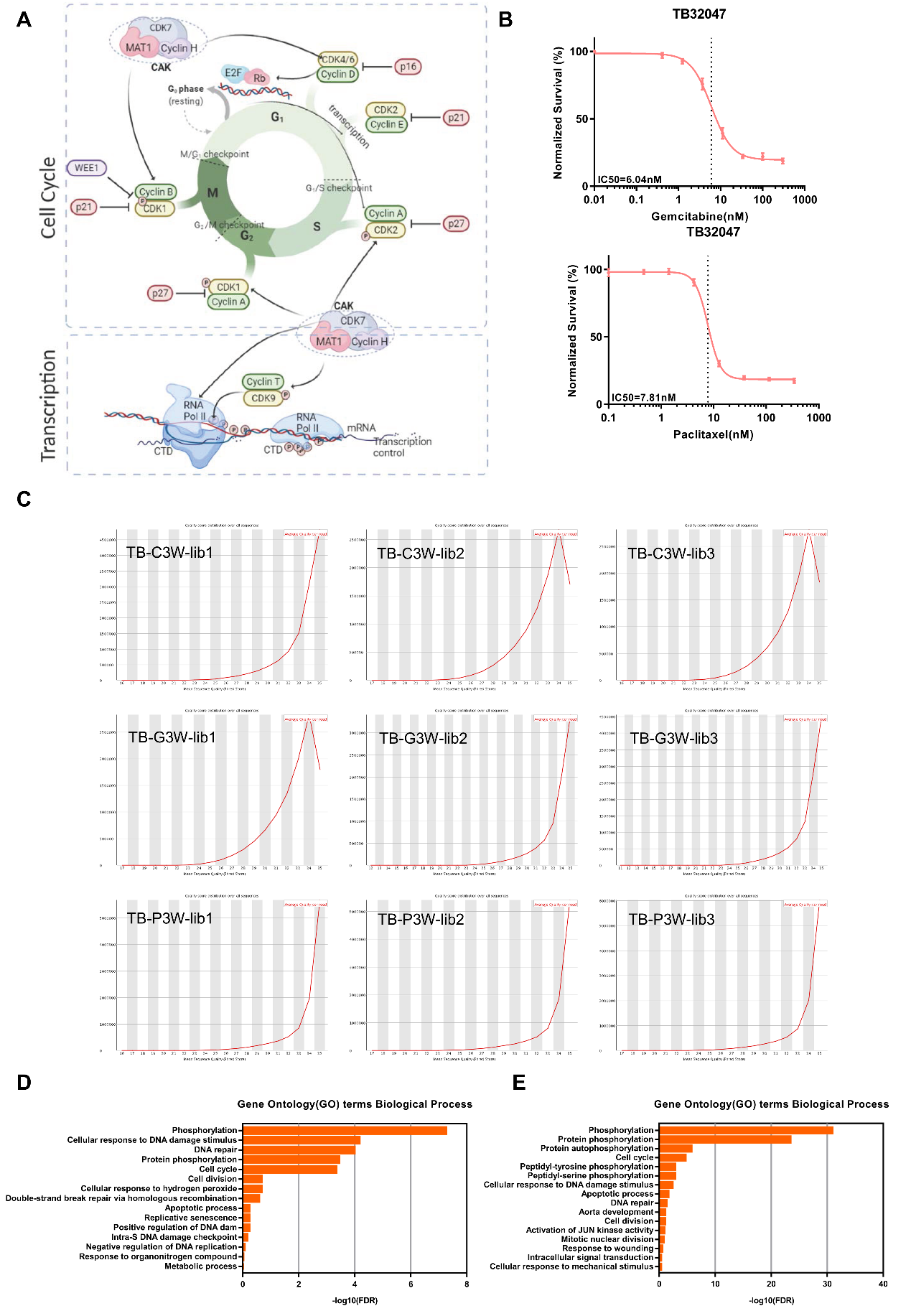
**

**Figure S1. A kinome-wide CRISPR screen in pancreatic cancer cells.**

**A.** Role of CDK7 in the regulation of cell cycle and transcription.

**B.** Dose–response curves of TB32047 cells treated for 72 hours with gemcitabine and paclitaxel. Data are presented as mean ± SD (n = 3).

**C.** MAGeCK-VISPR quality control analysis confirmed the mean sequence quality of each independent NGS data set. Each kinase library was run three times (n = 3).

**D.** Gene Ontology (GO) term biological process analysis of revealed pathway enrichment in the TB32047 gemcitabine screening results. Only statistically significant results (*p* < 0.05) are displayed.

**E.** Gene Ontology (GO) term biological process analysis of the TB32047 paclitaxel screening results revealed pathway enrichment. Only statistically significant results (*p* < 0.05) are presented.

**Figure S2.**

**
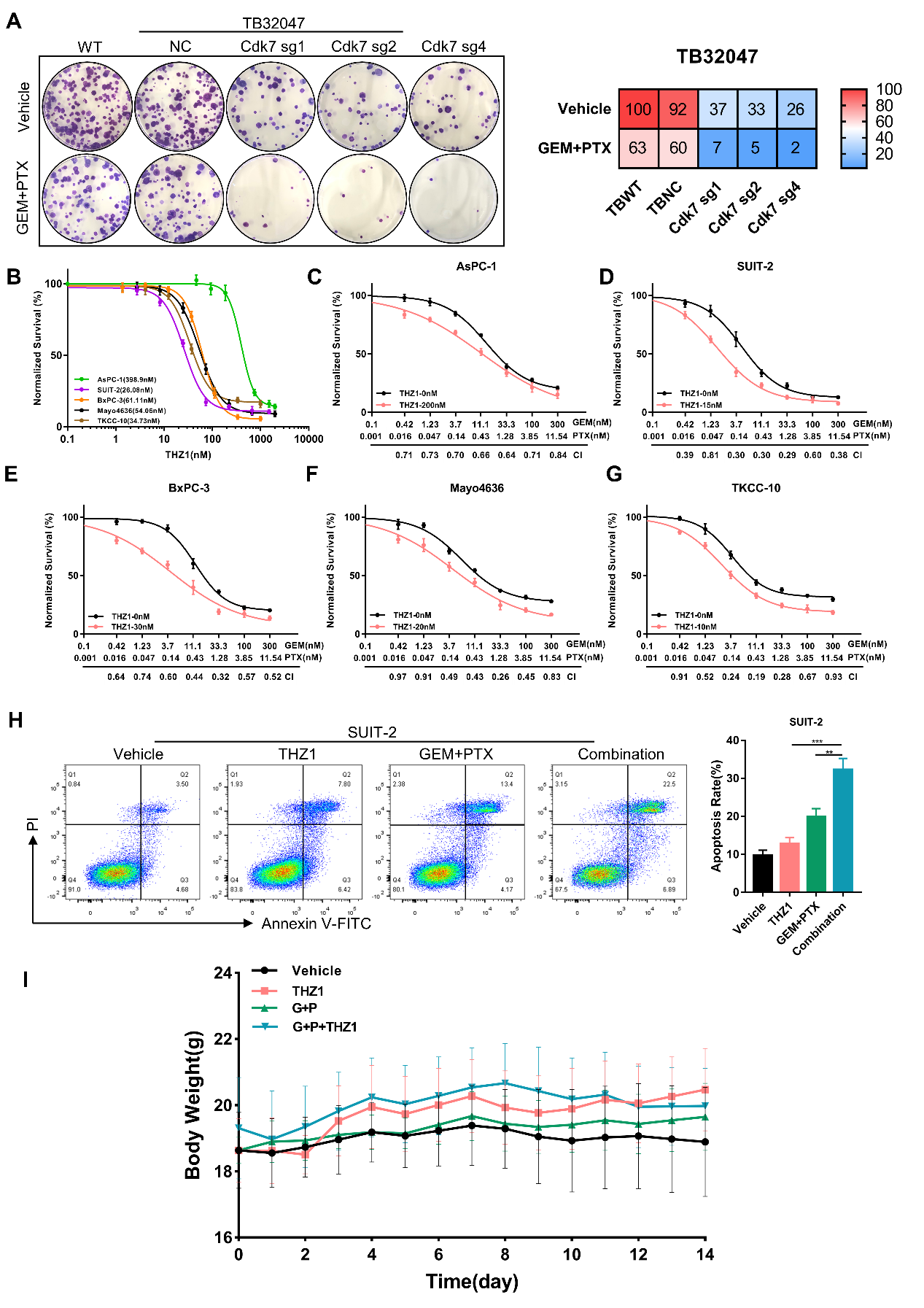
**

**Figure S2. Targeted inhibition of CDK7 enhances gemcitabine and paclitaxel chemotherapy response in pancreatic cancer in vitro and in vivo.**

**A.** Clonogenic assay utilizing vehicle-treated or gemcitabine-treated TB32047 Cdk7-KO cells. Representative figures (**left**) from three independent experiments (n = 3) are shown, as well as a heatmap depicting the proportion of live cells after treatment (**right**).

**B.** Dose–response curves for AsPC-1, SUIT-2, BxPC-3, Mayo4636, and TKCC-10 cells after 72 hours of treatment with THZ1. Data are presented as mean ± SD (n = 3).

**C-G.** Dose–response curves of AsPC-1 (**C**), SUIT-2 (**D**), BxPC-3 (**E**), Mayo4636 (**F**), and TKCC-10 (**G**) cells treated for 72 hours with THZ1 and GEM/PTX, alone and in combination. The combination index (CI) is depicted below the x-axis. CI < 1.0, synergism. Data are presented as mean ± SD (n = 3).

**H.** Apoptotic analysis of SUIT-2 cells after 24 hours of treatment with THZ1 and GEM/PTX, alone or in combination. Quantification (right) of three independent experiments (n = 3) are shown. ** *p* < 0.01 and *** *p* < 0.001 by one-way ANOVA with a Tukey multiple comparison test.

**I.** Body weights of TB32047 tumor-bearing mice during the treatment. Data are presented as mean ± SD (n = 5).

**Figure S3.
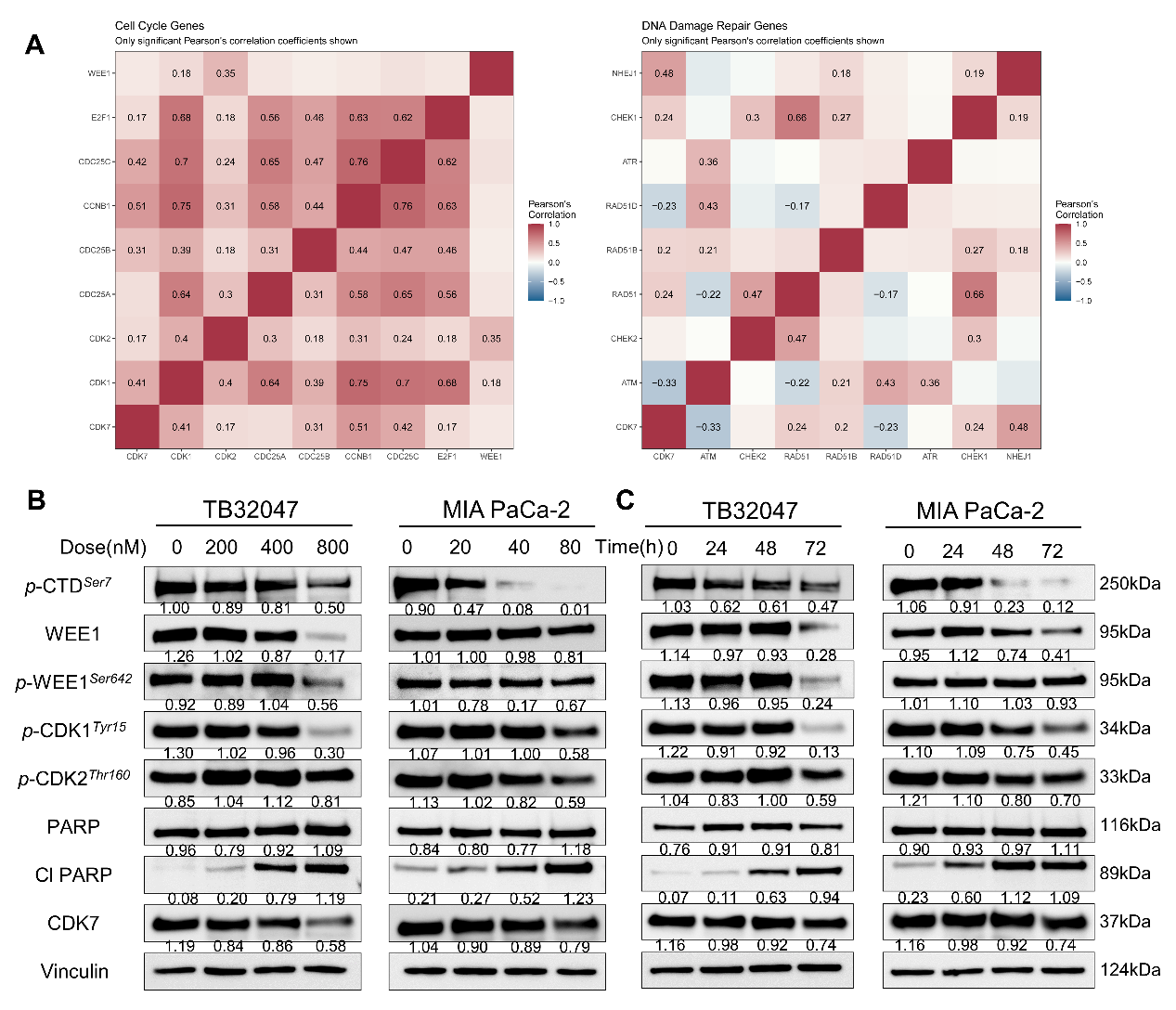

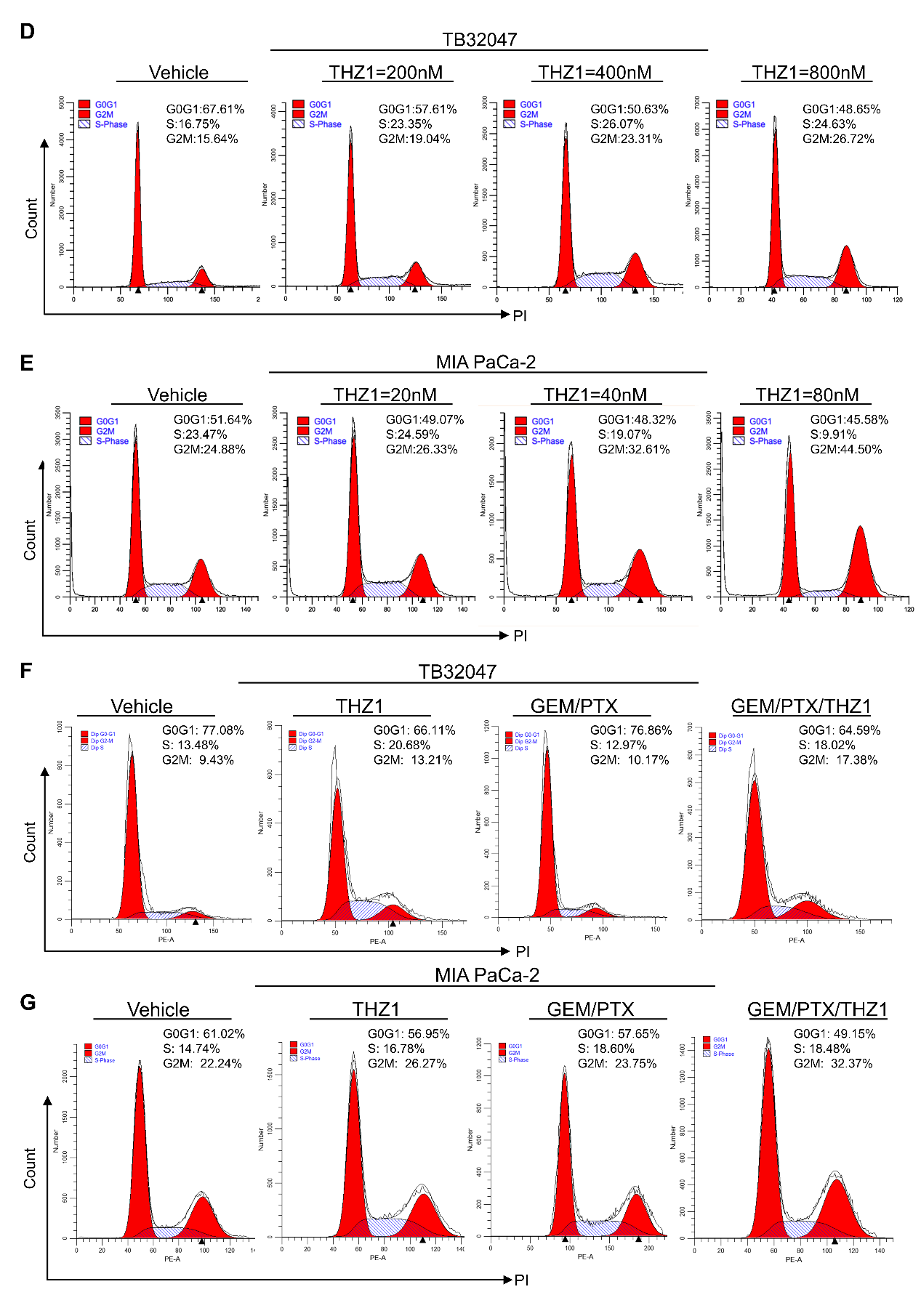
**

**Figure S3. CDK7 inhibition induces cell cycle arrest, apoptotic cell death, and DNA damage.**

**A.** CDK7 was correlated with the cell cycle gene panel and the DNA damage repair gene panel. Transcriptomic data of PDAC patients were downloaded from TCGA. Heatmap indicates Pearson's correlation coefficient. Significant positive (red) and negative (blue) correlations and only Pearson's correlation coefficients that were significant are shown, with color intensity proportional to the correlation coefficient. *p* < 0.05 was considered significant.

**B** **and C.** Western blotting of TB32047 and MIA PaCa-2 cells treated with different THZ1 concentrations (C) at different times (D) to ascertain the effects of CDK7 inhibition on cell signal regulation.

**D and E.** Cell cycle analysis: TB32047 (E) cells and MIA PaCa-2 (F) cells were treated variable concentrations of THZ1 for 24 hours. Representative figures from three independent experiments (n = 3) are shown.

**F and G.** Cell cycle analysis TB32047 (F) cells and MIA PaCa-2 (G) cells were treated with THZ1, GEM/PTX, or their combination for 24 hours. Representative figures from three independent experiments (n = 3) are shown.

**Figure S4.**


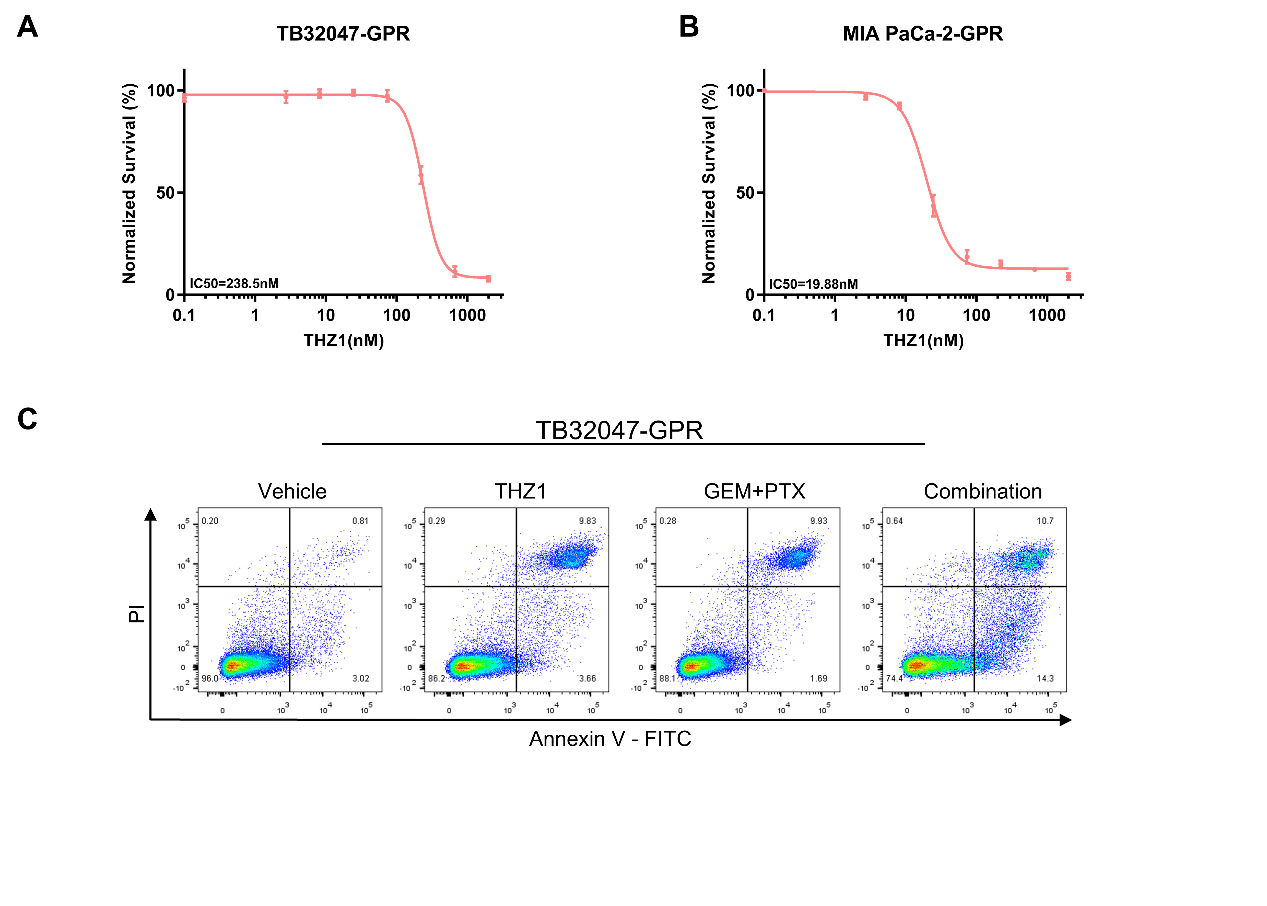


**Figure S4. Targeted inhibition of CDK7 reverses chemoresistance in pancreatic cancer.**

**A.** Dose–response curves of TB32047 gemcitabine- and paclitaxel-resistant cells treated for 72 hours with THZ1. Data are presented as mean ± SD (n = 3).

**B.** Dose–response curves of MIA PaCa-2 gemcitabine- and paclitaxel-resistant cells treated for 72 hours with THZ1. Data are presented as mean ± SD (n = 3).

**C.** Apoptotic analysis of TBGPR cells after 24 hours of treatment with THZ1, GEM+PTX, alone or in combination. Representative figures from three independent experiments (n = 3) are shown.

**Supplementary Tables**

**Table S1. Cell lines.**

**Table S2. Plasmids and primers list.**

**Table S3. Antibody list.**

**Table S4. List of** **CRISPR-Cas9 screening results of gemcitabine and paclitaxel in TB32047 cells.**
